# Workhouse Infirmaries and Consultants

**Published:** 1899-06-03

**Authors:** 


### WORKHOUSE INFIRMARIES AND CONSULTANTS.

We have received a copy of a resolution passed by a large meeting of medical men recently held in Bradford protesting against the proposal of the Bradford Board of Guardians to select medical officers for the workhouse infirmary by private treaty, and without inviting candidates to come forward by public advertisement, as being contrary to the general practice throughout the country, and detrimental to the interests of the public and of the medical profession. As an abstract resolution this seems to embody a very sound principle, and is one to which most people will be inclined to give a ready assent, for it is the system of open advertisement and free competition in every department which is concerned with the expenditure of public money that gives to the public the best possible guarantee against favouritism and abuse.

We fancy, however, that, although this lapse from a good principle is the particular detail in regard to which the medical profession of Bradford has chosen to criticise the action of the Guardians, there is a good deal behind which the resolution does not touch at all, and which is the real origin of the meeting and of the protest which it has brought forth.

It appears that on the retirement of Dr. Procter from the post of medical officer to the workhouse, the Guardians propose to appoint Dr. R. Crowley as his successor, Mr. R. Hall being added as a coadjutor. Now Dr. Crowley, who went to Bradford last year on his election as physician to the Bradford Royal Infirmary, is a consulting physician, and Mr. Hall is a consulting surgeon, and we fancy that we shall not be far from the truth if we say that the real question which is just now agitating the medical mind in Bradford has to do chiefly with the appointment of consultants, instead of general practitioners, to attend the patient at the workhouse. This is, of course, a large question, and involves many considerations. The great danger which overhangs all attempts to introduce the consulting element into workhouse infirmaries lies in the probability that before long the Guardians, having in many other ways assimilated their infirmaries to general hospitals, will seek to go a step further and will endeavour to obtain the services of an honorary medical staff. Probably this danger is not as great a one as some imagine, still it is one that deserves careful consideration. But if this could be eliminated—and surely if the profession has any power of combination left at all such a danger ought to be capable of being reduced within very small dimensions—then we could hardly avoid the conclusion that the introduction of the consulting element, together with the appointment of properly qualified resident medical officers to the large workhouse infirmaries which are growing up in our great towns, would be a great step forwards. When, however, we say the consulting element, we would guard ourselves against misconception. We mean that there should be properly paid visiting surgeons and physicians, and that the details of the treatment should be carried out by residents. But we by no means tie ourselves down to the suggestion that the visiting officers should be pure consultants. They should just simply be the best men that can be got to do the work. Here, then, we are in sympathy with the resolution passed by the medical men of Bradford, for we cannot believe that private treaty is the best way to get the best men. But if, as an *arrière pensée*, the medical men of Bradford cherish a dislike to the idea of the workhouse infirmary being officered by visiting physicians and surgeons and by a resident medical staff, and if that is the ground of their action, then we must differ from them.

